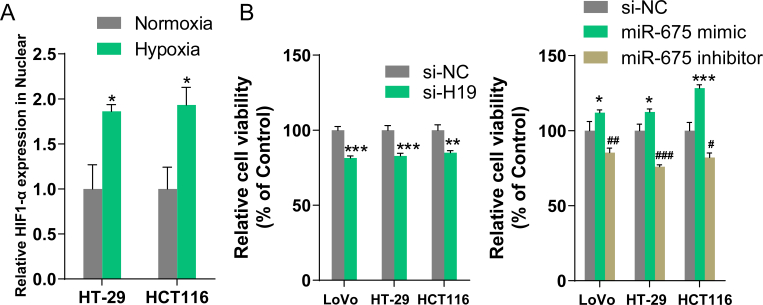# Decreased expression of H19/miR-675 ameliorates hypoxia-induced oxaliplatin resistance in colorectal cancer

**DOI:** 10.1016/j.heliyon.2024.e27027

**Published:** 2024-02-28

**Authors:** Xingyue Weng, Tao Ma, Qi Chen, Bryan Wei Chen, Jianzhen Shan, Wei Chen, Xiao Zhi

**Affiliations:** a Department of Medical Oncology, The First Affiliated Hospital, Zhejiang University School of Medicine, No.79, Qingchun Road, Hangzhou, Zhejiang, 310003, China; b Department of Hepatobiliary and Pancreatic Surgery and Zhejiang Provincial Key Laboratory of Pancreatic Disease, The First Affiliated Hospital, Zhejiang University School of Medicine, No.79, Qingchun Road, Hangzhou, Zhejiang, 310003, China; c Cancer Institute of Integrated Traditional Chinese and Western Medicine, Zhejiang Academy of Traditional Chinese Medicine, Tongde Hospital of Zhejiang Province, Hangzhou, 310012, Zhejiang Province, China

## Abstract

Hypoxic microenvironment, a hallmark of solid tumors, contributes to chemoresistance, and long noncoding (lnc) RNAs are involved in hypoxia-induced drug resistance. However, the role of lncRNAs in hypoxic tumor chemotherapy resistance remains unclear. Here, we aimed to elucidate the effects of lncRNAs in hypoxia-mediated resistance in colorectal cancer (CRC), as well as the underlying mechanisms. The results indicated that the expression of lncRNA H19 was enhanced in hypoxia- or oxaliplatin-treated CRC cells; moreover, H19 contributed to drug resistance in CRC cells both in vitro and in vivo. Mechanistically, H19 was noted to act as a competitive endogenous RNA of miR-675-3p to regulate epithelial–mesenchymal transition (EMT). Notably, an miR-675-3p mimic could attenuate the effects of H19 deficiency in CRC cells with hypoxia-induced chemoresistance. In conclusion, H19 downregulation may counteract hypoxia-induced chemoresistance by sponging miR-675-3p to regulate EMT; as such, the H19/miR-675-3p axis might be a promising therapeutic target for drug resistance in CRC.

## 1 Introduction

Colorectal cancer (CRC) is one of the most common malignancies of the digestive system globally. As of 2020, CRC demonstrated the third-highest incidence and second-highest mortality among all cancers globally [1]. Surgery, chemotherapy, and radiotherapy are currently the main CRC treatment methods. However, CRC prognosis remains poor, with the overall 5-year survival rate being <15% [2]. Surgery remains the preferred first-line treatment for CRC detected early [3]. However, in most patients, CRC is diagnosed at later stages and typically treated with chemotherapy. Currently, platinum-based adjuvant chemotherapy is the standard treatment modality for stage III colon cancer [4]. However, long-term use of platinum-based drugs may lead to drug resistance and serious side effects. Therefore, the discovery and development of newer treatment methods for improving the chemical sensitivity of CRC to platinum-based drugs with minimized adverse effects, improved treatment efficacy, prolonged disease-free survival, and improved prognosis are necessary.

A low-oxygen microenvironment is one of the most significant features of solid tumors. Tumors demonstrate abnormal vasculature and fast proliferation. Therefore, at tumor sites, oxygen consumption is greater than oxygen supply; this leads to the formation of a hypoxic microenvironment at these sites. Hypoxia can cause genomic changes, enabling tumor cells to adapt to poor nutrition and adverse microenvironments, thereby facilitating the maintenance of vitality [5]. Hypoxia is important for tumor occurrence and progression, specifically chemotherapy resistance promotion [6,7]. A hypoxic microenvironment can promote chemotherapy resistance in tumor cells through various related mechanisms, including promoting epithelial–mesenchymal transition (EMT), a mechanism leading to chemotherapy resistance in tumors [[8], [9], [10]]. Therefore, strategies for regulating hypoxia-mediated chemotherapy resistance in CRC have broad clinical potential.

Long noncoding RNAs (lncRNAs) are >200-nucleotide-long RNAs with no protein-coding potential [11,12]. They can form RNA–RNA, RNA–DNA, and RNA–protein complexes to regulate gene expression through various mechanisms, including transcriptional regulation, mRNA stability, and translation [13,14]. Abnormal expression levels of lncRNAs may be closely related to malignant tumor occurrence, metastasis, and drug resistance [15,16]. Many lncRNAs expressed differentially in response to hypoxia play a critical role in cancer development [17]. For instance, silencing of lncRNA H19 against hypoxia-induced injury in PC-12 cells by regulating miR-28 expression [18]. H19 participates in hypoxia-driven migration and invasion in glioblastoma cells via regulation miR-181d [19]. Hypoxia upregulates the expression of lncRNA H19 in non-small cell lung cancer cells and induces drug resistance [20]. In addition, miRNAs also play an important role in hypoxic resistance. Hypoxia-Induced miR-675-5p could Support β-Catenin nuclear localization through regulation of the acitivition of GSK3-β in colorectal cancer cells [21]. MiR-675-3p is upregulated in melanoma cell lines and may activate TGF-beta and HIF-1 signaling [22]. It has been reported that miR-27a was involved in gastric cancer hypoxia-induced chemoresistance [23] However, the role of lncRNAs and miR-675-3p in hypoxia signaling, particularly in hypoxic tumor chemotherapy resistance, remains elusive.

In this study, we observed changes in a group of hypoxia-associated lncRNAs before and after hypoxia or oxaliplatin treatment. Of them, high expression of the lncRNA H19 was noted to confer oxaliplatin resistance both in vitro and in vivo. Mechanistically, H19 acted as a competitive endogenous RNA of miR-675-3p, thus regulating EMT.

## 2 Materials and methods

### 2.1 Cell culture

LoVo, HT29, and HCT-116 cells were cultured in F-12 K, RPMI-1640, and McCoy's 5A media, respectively; all media were supplemented with 10% fetal bovine serum (Gibco). The incubation conditions were 37 °C under 5% CO2. During hypoxia treatment, cells were cultured with 5% CO2, 1% O2, and 94% N2 for 12 h.

### 2.2 RNA extraction and reverse transcription quantitative polymerase chain reaction analysis

Total RNA was extracted by lysing cells by using TRIzol (Takara, Dalian, China) and then reverse-transcribed to cDNA for H19 and miR-675 using a PrimeScript RT reagent Kit with gDNA Eraser (Perfect Real Time; Takara) or a Mir-X microRNA (miRNA) First-Strand Synthesis Kit (Takara), respectively. Next, reverse transcription quantitative polymerase chain reaction (RT-qPCR) was performed using SYBR Green Mix (Takara) using an ABI 7500 system (Applied Biosystems). *GAPDH* and *U6* were used as internal controls for H19 and miR-675-3p, respectively. Relative expression levels were calculated using the 2^−ΔΔCq^ method.

### 2.3 Cell transfection

MiR-675-3p mimics, miR-675-3p inhibitors, and miRNA-negative control (miR-NC) were purchased from RiboBio (Guangzhou, China). Specific small interfering RNAs (siRNAs) targeting H19 (si-H19) and negative control (si-NC) were designed and synthesized by Genepharm (Shanghai, China). The CRC cells were transfected with equal concentrations of oligo fragments diluted in Opti-MEM medium (Gibco) by using Lipofectamine 2000 (Invitrogen, Carlsbad, CA, USA) at approximately 80% confluence, according to the manufacturer's instructions.

### 2.4 CCK-8 assay

Cell proliferation was analyzed using a CCK-8 assay kit (Dojindo Molecular Technologies), according to the manufacturer's instructions. In brief, transfected cells were seeded into 96-well plates at 5 × 10^3^ cells/well, followed by incubation for 48 h. Next, the supernatant was aspirated, and culture medium (100 μL) was added to each well. CCK-8 reagent (10 μL/well) was added to each well, followed by incubation at 37 °C for 2 h. The optical density (OD) values were detected at a wavelength of 450 nm under a microplate reader (Bio-Rad).

### 2.5 Ethynyl-2′-deoxyuridine assay

CRC cell proliferative ability was detected using 5-ethynyl-2′-deoxyuridine (EdU) staining (Thermo Fisher Scientific), according to the manufacturer's instructions. The CRC cell lines were seeded in 96-well plates at a density of 5 × 10^3^ cells/well in correspondence medium. The medium was replaced with the corresponding serum-free medium to synchronize the cells. After 24 h, the serum-free medium was replaced with growth media containing drugs at the indicated concentrations for 48 h. At last, cells were counterstained with Hoechst 3342 and DAPI.

### 2.6 Western blot analysis

Cells were lysed to extract total protein with radioimmunoprecipitation assay lysis buffer (RIPA) buffer (Beyotime). Equal amounts of protein were separated through 10% sodium dodecyl sulfate polyacrylamide gel electrophoresis, followed by transfer onto polyvinylidene difluoride membranes. These membranes were then blocked in 3% bovine serum albumin for 2 h and then incubated with primary antibodies against E-cadherin (1:1000), N-cadherin (1:1000), and GAPDH (1:2000; all from Abcam, Cambridge, MA, USA) at 4 °C overnight. The membranes were then incubated with horseradish peroxidase–conjugated secondary antibodies at room temperature for 2 h. Protein bands were then developed using enhanced chemiluminescence reagent (Beyotime) and quantified using ImageJ (National Institutes of Health, Bethesda, MD, USA).

### 2.7 Nuclear fraction analysis

Nucleocytoplasmic separation Nuclear and cytosolic fractions were separated using the PARIS kit (Thermo Fisher Scientific, USA) according to manufacturer's protocol. Later, the levels of GAPDH, U1 and H19 in cytoplasm or nuclear of LoVo cells were examined using qRT-PCR assay.

### 2.8 Immunofluorescence assay

The CRC cells were inoculated on cell slides and subjected to immunofluorescence assay. The cells were first fixed using 4% paraformaldehyde for 15 min and washed three times with PBS. Subsequently, the slides were immersed in a blocking buffer (0.3% Triton X-100) for 60 min, incubated with relevant primary antibodies (1:100 dilution; CST) at 4 °C overnight, and treated with fluorochrome-conjugated secondary antibodies (1:100 dilution) at 37 °C for 1 h. After the nuclei were stained with DAPI for 30 min, the slides were analyzed under a fluorescence microscope (Olympus).

### 2.9 Tumor xenografts in nude mice

The animal study was conducted in accordance with the National Institutes of Health Guide for Care and Use of Laboratory Animals and approved by the Medical Ethics Committee of the First Affiliated Hospital of Zhejiang University.

We injected 1 × 10^6^ HT-29 cells subcutaneously into the lateral flank of 6-week-old BALB/c nude mice to form tumors. The tumors were then sectioned at 1 mm^3^ and embedded in subcutaneous tissues. The mice were divided into four groups (n = 6 per group): normal saline, oxaliplatin, normal control plus oxaliplatin, and H19 shRNA plus oxaliplatin. To introduce H19 shRNA, 50 μL of shRNA H19 lentivirus at a concentration of 1E8/mL was injected into the tumor at the appropriate volume on days 1 and 8. Oxaliplatin was injected into the tail vein at 8.3 mg/kg. The tumor volume was measured every other day and calculated using the following formula:

$$\text{Tumor}\;\text{size}=\left({\text{width}}^{2}\times \text{length}\right)/2$$

### 2.10 TUNEL assay

Apoptotic cells were detected using a TUNEL kit (Thermo Fisher Scientific), according to the manufacturer's instructions. Briefly, paraffin-embedded sections were deparaffinized and hydrated in a graded ethanol series and then digested with trypsin for 40 min at room temperature, and then were incubated with TUNEL reaction buffer in a 37 °C for 1 h, washed with PBS. The apoptotic cells were observed under a light microscope (Olympus, Tokyo, Japan).

### 2.11 Statistical analysis

Data are expressed as mean ± standard deviation (SD). Student's *t*-test was used to compare two groups. *P* < 0.05 was considered to indicate statistical significance.

## 3 Results

### 3.1 H19 expression is enhanced in hypoxia- or oxaliplatin-treated CRC cells

**Fig. 1:**
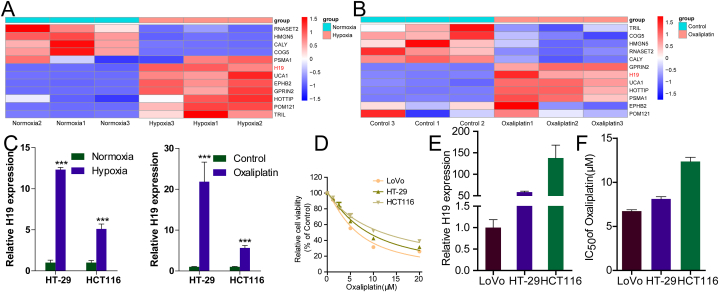
H19 expression is enhanced in hypoxia- or oxaliplatin-treated CRC cells. **(A)** RT-qPCR for changes in lncRNA expression in LoVo cells under hypoxia. **(B)** RT-qPCR for changes in lncRNA expression in LoVo cells under oxaliplatin treatment. **(C)** RT-qPCR for H19 expression in hypoxia- or oxaliplatin-treated CRC cells. **(D)** CCK-8 assay for cell viability after oxaliplatin treatment; IC50 of each group presented in a bar chart. **(E)** RT-qPCR for H19 expression in CRC cells. (F) The value of IC50 in CRC cell lines.

First, we selected a group of lncRNAs reported to be associated with hypoxia and detected the changes in their expression in LoVo cells before and after hypoxia or oxaliplatin treatment by using RT-qPCR (Fig. 1A and B). The results demonstrated that H19 was significantly upregulated under both hypoxia and oxaliplatin treatment.

Next, we confirmed these results in two other CRC cell lines, HCT116 and HT29. Our RT-qPCR results demonstrated that H19 expression was higher in a hypoxic environment or under oxaliplatin treatment than in the corresponding control groups (Fig. 1C). We also detected the nuclear level of HIF-1α in CRC cells, showed that the level of HIF-1α was up-regulated in CRC cells (Supplementary Fig. 1 A). These data indicated that H19 expression is enhanced in hypoxia- or oxaliplatin-treated CRC cells.

Next, we compared the sensitivities of different CRC cell lines to oxaliplatin; it demonstrated the highest half maximal inhibitory concentration (IC50) in HCT116 cells, followed by that in HT29 and LoVo cells (Fig. 1D). We also compared H19 expression among the three CRC cell lines. H19 expression was the highest in HCT116 cells, followed by HT29 cells and then by LoVo cells (Fig. 1E). **The IC50 of Oxaliplatin in CRC cell lines was shown in Fig. F**. This result was consistent with the result for IC50, suggesting that the increase in H19 expression might be related to oxaliplatin resistance.

### 3.2 H19 silencing attenuates oxaliplatin resistance in CRC cells

**Fig. 2:**
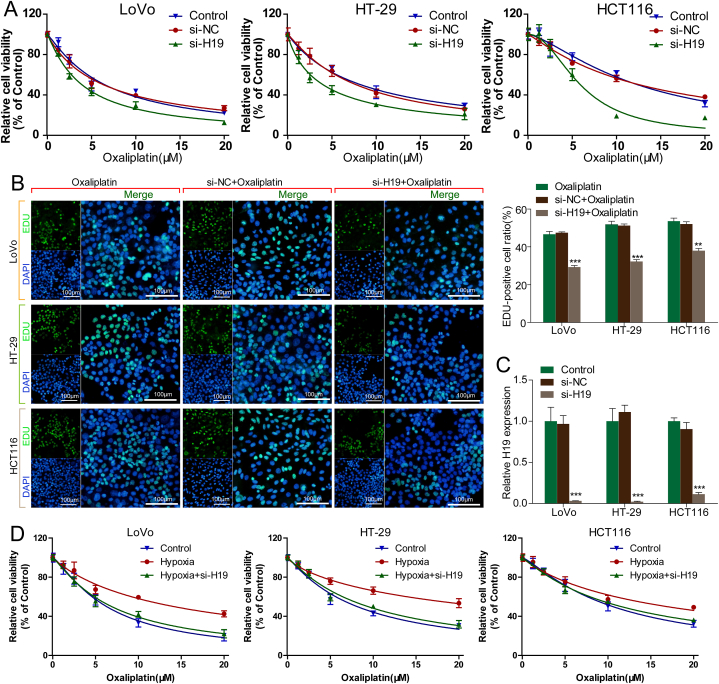
H19 deletion attenuates oxaliplatin resistance in CRC cells under normoxia and hypoxia. **(A)** Relative cell viability in oxaliplatin-treated CRC cells transfected with si-NC and si-H19. **(B)** Cell proliferative viability in oxaliplatin-treated CRC cells transfected with si-NC and si-H19, with EdU-positive cell ratio. **(C)** H19 expression in CRC cells transfected with si-H19 or si-NC. **(D)** Relative cell viability in oxaliplatin-treated CRC cells transfected with si-H19 or si-NC under normoxia or hypoxia.

To determine whether H19 participates in oxaliplatin resistance development in CRC cells, si-H19 was transfected into CRC cells to knock down H19 expression. Firstly, we used RT-qPCR to confirm the efficiency of H19 siRNA, the results showed that si-H19 transfection inhibited H19 expression (Fig.2C). Later, cell viability assay results revealed that H19 knockdown significantly enhanced cytotoxicity due to oxaliplatin in CRC cells cultured under normoxia (Fig. 2A). Moreover, our cell proliferation assay result demonstrated that CRC cells exhibited a lower new DNA synthesis rate in the si-H19 group than in the si-NC group, reflected by a decrease in the EdU-positive cell rate after 48 h of culture with oxaliplatin (Fig. 2B).

Next, we investigated the role of H19 knockdown in hypoxia-induced chemoresistance. Our cell viability assay results revealed that the relative viability of CRC cells was significantly higher under hypoxia than under normoxia. Moreover, CRC cells were significantly more sensitive to oxaliplatin under hypoxia in the si-H19 group than in the si-NC group (Fig. 2D). These results indicated that H19 knockdown increases oxaliplatin toxicity in CRC cells.

### 3.3 H19 knockdown enhances oxaliplatin efficacy in vivo

**Fig. 3:**
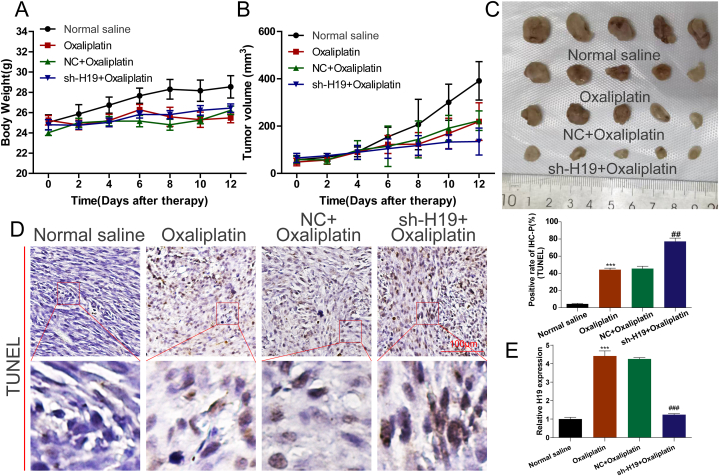
H19 knockdown enhances oxaliplatin treatment efficacy in xenograft tumors. **(A)** Body weight in nude mice with different treatments. **(B)** Tumor volume in nude mice with different treatments. **(C)** Representative images of xenograft tumors after 2 weeks. **(D)** Apoptosis in nude mice with different treatments determined by TUNEL assay; lower panels include amplified images of the red frames of upper panels. (E) RT-qPCR determined the level of H19 in different treatment group (Normal Saline, Oxaliplatin, NC + Oxaliplatin, sh-H19 + Oxaliplatin).

To investigate the role of H19 in oxaliplatin resistance in vivo, a xenograft model was established, and lentivirus-sh-H19 was injected intratumorally. The body weight was similar among all four treatment groups (Fig. 3A). Compared with normal saline, oxaliplatin considerably reduced tumor volume, which was further reduced by sh-H19 (Fig. 3B and C). Moreover, compared with normal saline, oxaliplatin increased apoptosis, reflected by the TUNEL-positive rate, which increased further after H19 knockdown (Fig. 3D). We also determined the level of H19 in different treatment group(Normal Saline, Oxaliplatin, NC + Oxaliplatin, sh-H19 + Oxaliplatin), the results indicated that sh-h19 + Oxaliplatin treatment could down-regulated the expression of H19 comparison with NC + Oxaliplatin group(Fig. 3 E).

### 3.4 H19 deletion partially blocks hypoxia-induced EMT

**Fig. 4:**
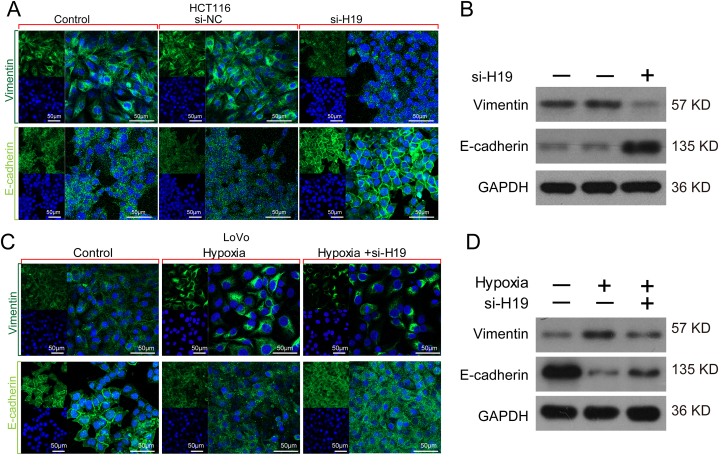
H19 deletion partially blocks hypoxia-induced EMT. **(A)** Immunofluorescence analysis of EMT-related proteins in HCT116 cells transfected with si-NC and si-H19. **(B)** Western blot analysis of E-cadherin and vimentin in HCT116 cells transfected with si-NC and si-H19. **(C)** Immunofluorescence analysis of EMT-related proteins in LoVo cells transfected with si-NC and si-H19 under hypoxia or normoxia. **(D)** Western blot analysis of E-cadherin and Vimentin in LoVo cells transfected with si-NC and si-H19 under hypoxia or normoxia.

EMT has become a hot topic in recent cancer research. EMT is a cellular activity transformation characterized by E-cadherin downregulation, as well as upregulation of mesenchymal phenotype markers such as vimentin. It is crucial in the development of not only tumor cell infiltration and metastasis under hypoxia but also chemotherapy resistance. Here, we used immunofluorescence assay and Western blot analysis to evaluate whether H19 affects E-cadherin and vimentin expression in CRC cells. The results revealed that E-cadherin expression was significantly increased after H19 silencing in HCT116 cells than in control cells, accompanied by a significant decrease in vimentin expression during the EMT process (Fig. 4A and B). Notably, hypoxia reduced E-cadherin expression and increased vimentin expression in LoVo cells than in control cells, but H19 deletion reversed hypoxia-induced EMT (Fig. 4C and D).

### 3.5 MiR-675-3p is a key regulator in H19-mediated oxaliplatin sensitivity in CRC cells

LncRNAs demonstrate different cellular localizations; as such, their functions also differ completely. Therefore, we first detected the cellular localization of H19 before and after hypoxia through fluorescence in situ hybridization (FISH). The results demonstrated that H19 is mainly expressed in the cytoplasm.

**Fig. 5:**
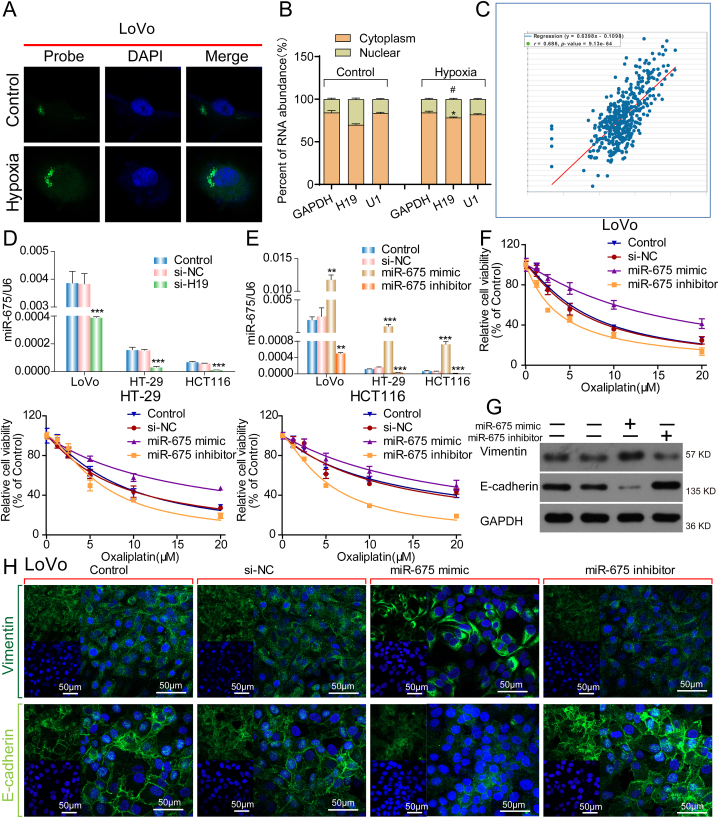
miR-675-3p is a key regulator in H19-mediated oxaliplatin sensitivity in CRC cells. **(A)** Immunofluorescence staining for subcellular localization of H19 in LoVo cells under hypoxia or normoxia. (B) RT-qPCR analysis was performed to determine the relative distribution of H19 in nuclear and cytoplasm of LoVo cells. (C) Correlation between mir-675 and H19 by Starbase. (D) RT-qPCR for miR-675-3p expression in CRC cells after H19 knockdown. (E) RT-qPCR for miR-675-3p expression levels in CRC cells after control (miR-NC), miR-675-3p mimic, or miR-675-3p inhibitor transfection. (F) Relative viability in nontransfected CRC cells or CRC cells transfected with an miR-675-3p mimics or inhibitors after oxaliplatin treatment. (G) Western blot analysis of E-cadherin and Vimentin in LoVo cells transfected with an miR-675-3p mimics or inhibitors. (H) Immunofluorescence analysis of EMT-related proteins in HCT116 cells transfected with an miR-675-3p mimics or inhibitors.

In the cytoplasm, lncRNAs mainly exert their function by interacting with miRNAs as sponges or precursors [24]. Therefore, after confirming that H19 is located in the cytoplasm (Fig. 5A), we further detected the relative distribution of H19 in nuclear and cytoplasm of LoVo cells, showing that the percent of H19 RNA abundance was increased under hypoxia(Fig. 5B). Starbase was used to predicate the relationship between H19 and miR-675-3p, indicated that miR-675-3p was noted to be closely correlated with H19 (Fig. 5C). H19 knockdown significantly reduced miR-675-3p expression (Fig. 5D).

To determine whether miR-675-3p is involved in oxaliplatin resistance in CRC cells, the CRC cells were transfected with an miR-675-3p mimic or inhibitor to alter miR-675-3p expression. Our RT-qPCR results confirmed the efficiency of transfection (Fig. 5E). Moreover, our CCK-8 assay results revealed that in CRC cells, miR-675-3p inhibitors significantly enhanced oxaliplatin toxicity, whereas miR-675-3p mimics reduced it (Fig. 5F). Moreover, our Western blot analysis results demonstrated that miR-675-3p mimics increased vimentin protein expression but reduced E-cadherin expression, whereas miR-675-3p inhibitors had the exact opposite effect (Fig. 5G). Our immunofluorescence assay results were consistent with those of our Western blot analysis (Fig. 5H). Taken together, these results indicated that H19 might increase the oxaliplatin sensitivity of CRC cells through miR-675-3p.

### 3.6 H19 knockdown enhances oxaliplatin sensitivity through miR-675-3p regulation

**Fig. 6:**
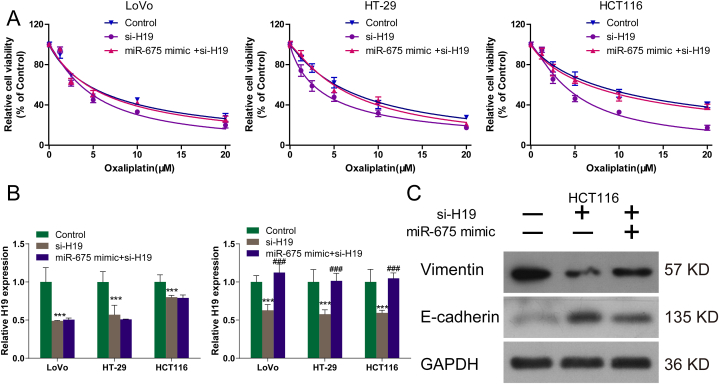
H19 knockdown enhances oxaliplatin sensitivity through miR-675-3p regulation. (A) Relative viability of oxaliplatin-treated CRC cells transfected with si-H19 alone or with an miR-675-3p mimic. (B) The transfection of si-H19 and miR-675-3p were confirmed by RT-qPCR. (C) Western blot analysis of E-cadherin and vimentin in HCT116 cells transfected with si-H19 alone or with an miR-675-3p mimic.

To investigate whether miR-675-3p mediates the effects of H19 in oxaliplatin resistance, H19-knockdown CRC cells were transfected with or not transfected with miR-675-3p mimics. The transfection of si-H19 and miR-675-3p was determined by RT-qPCR (Fig.6B). Transfection with miR-675-3p mimics partially rescued the effects of H19 knockdown on oxaliplatin resistance and EMT in HCT116 cells, respectively (Fig. 6A and C, respectively).

## 4 Discussion

CRC remains a major cause of cancer-related deaths, and tumor resistance poses a significant challenge in CRC therapy. The current results indicated that the lncRNA H19 is overexpressed in CRC cells under hypoxia or treated with oxaliplatin. H19 knockdown attenuated oxaliplatin resistance in CRC cells under both normoxia and hypoxia. Furthermore, H19 knockdown significantly improved the efficacy of oxaliplatin in xenograft tumors. Finally, we noted that the H19/miR-675-3p axis contributed to hypoxia-induced resistance in CRC cells. Therefore, H19 is a potential therapeutic target in CRC.

Drug resistance is a complex phenomenon involving multiple mechanisms. Accumulating evidence suggests that hypoxia within tumors contributes to the development of resistance to radiotherapy and chemotherapy [25]. Moreover, hypoxia-induced noncoding RNAs have a crucial role in the mediation of hypoxia response (including drug resistance) [[26], [27], [28], [29]]. It has been reported that lncH19 is among the hypoxia-induced lncRNA well characterized in CRC [30]. In this study, we selected a group of previously reported hypoxia-related lncRNAs and assessed changes in their expression in CRC cells before and after oxaliplatin treatment or hypoxia exposure. We noted that H19 was significantly upregulated under hypoxia or oxaliplatin treatment. H19 can enhance SIRT1-mediated autophagy-mediated 5-fluorouracil resistance in colon cancer [31]. Cancer-associated fibroblasts promote colon cancer stemness and chemoresistance via H19 secretion [32]. Sun et al. used bioinformatics analysis and found that four lncRNAs, including H19, were predictive factors for sensitivity to CRC chemotherapy [33]. Similarly, we found that H19 knockdown attenuated oxaliplatin resistance in CRC cells and partially reversed hypoxia-induced drug resistance. Moreover, we found that treatment with H19 siRNA or miR-675-3p alone, the cell viability was no significate difference between control and H19 siRNA, or Control and miR-675-3p siRNA (Supplementary Fig. 1 B).

LncRNAs have been identified to participate in various cellular processes, including proliferation, apoptosis, migration, and invasion. They regulate the epigenetic, transcriptional, and posttranscriptional expression levels of genes depending on their cellular localization. Notably, these lncRNAs are involved in tumor chemoresistance regulation through various mechanisms. Inhibition of lncRNA TTN-AS1 could regulate osteosarcoma cell apoptosis and drug resistance via the miR-134-5p/MBTD1 axis [34]. Knockdown of CCAT1 could inhibit cell proliferation and enhance drug sensitivity by regulation miR-143/PLK1/BUBR1 signaling [35]. H19 also could regulate drug resistance in various cancer cells. For instance, H19 could regulate cell proliferation and cell metastasis by miR-29b-3p/PGRN/EMT axis and Wnt signaling [36]. H19 could promote lung adenocarcinoma cells viability and EMT of by targeting miR-29b-3p and modifying STAT3 [37]. Knockdown of H19 could increase the chemosensitivity of GC cells to ADM via sponging miR-152 from TCF4 and EMT [38]. In this study, we further confirmed that si-H19 could enhance oxalipatin sensitivity by EMT, we also determined the localization of H19 in CRC cells through FISH: H19 is mainly expressed in the cytoplasm. LncH19 exon one encodes for two different miRNAs, miR-675-3p, and through it controls most of the tumor processes in which it is involved [39]. Several miRNAs may play a role in chemoresistance development. Through database comparison, we found that miR-675-3p (mature miRNAs produced from H19) had the strongest correlation with H19. Studies have shown that H19 and miR-675-3p expression are aberrant in many tumors, indicating their strong association with tumor initiation and progression. For instance, in various cancers such as squamous cell carcinoma of the skin [40], gastric cancer [41], pancreatic cancer [42], and nasopharyngeal carcinoma [43], H19 and miR-675-3p expression is significantly upregulated. In this study, we found that miR-675-3p inhibition attenuates oxaliplatin resistance in CRC cells. MiR-675-3p mimic transfection partially rescued the effects of stable H19 knockdown on oxaliplatin resistance in CRC cells.

## 5 Conclusions

In summary, the lncRNA H19 increases drug resistance in hypoxia- or oxaliplatin-treated CRC cells. H19 acts as a competitive endogenous RNA against miR-675-3p to regulate EMT. This H19/miR-675-3p axis might be a promising therapeutic target for CRC resistance.

## Ethics approval and consent to participate

All animal experiments were approved by the First Affiliated Hospital of Zhejiang University of Medical Ethics Committee and the Medical Faculty Ethics Committee of the First Affiliated Hospital of Zhejiang University in accordance with the National Institutes of Health Guide for Care and Use of Laboratory Animals (NIH Publications, No. 8023, revised 1978).

## Consent to publication

All authors have consented to the publication of this manuscript here.

## Funding

This work was supported by the 10.13039/501100001809National Natural Science Foundation of China (grant number 82171721, 81702376 and 82071748) and 10.13039/501100004731Zhejiang Provincial Natural Science Foundation of China under Grant No. LY21H160021.

## Data availability statement

We declare that all data support the conclusions of the study and all data will be made available on request.

## CRediT authorship contribution statement

**Xingyue Weng:** Methodology, Formal analysis, Data curation. **Tao Ma:** Funding acquisition, Formal analysis, Data curation. **Qi Chen:** Methodology, Data curation. **Bryan Wei Chen:** Data curation. **Jianzhen Shan:** Methodology, Formal analysis. **Wei Chen:** Writing – original draft, Conceptualization. **Xiao Zhi:** Writing – review & editing, Funding acquisition, Conceptualization.

## Declaration of competing interest

The authors declare the following financial interests/personal relationships which may be considered as potential competing interests:Wei Chen reports was provided by National Natural Science Foundation of China. Wei Chen reports a relationship with National Natural Science Foundation of China that includes:. Wei Chen has patent pending to Wei Chen. The authors declare that they have no competing interests regarding the content of this article. Wei Chen If there are other authors, they declare that they have no known competing financial interests or personal relationships that could have appeared to influence the work reported in this paper.

## Figures and Tables

**figs1:**